# Development of a simple mechanical measurement method to measure spasticity based on an analysis of a clinical maneuver and its concurrent validity with the modified Ashworth scale

**DOI:** 10.3389/fbioe.2022.911249

**Published:** 2022-08-15

**Authors:** Hiroki Tanikawa, Masahiko Mukaino, Shota Itoh, Hikaru Kondoh, Kenta Fujimura, Toshio Teranishi, Kei Ohtsuka, Satoshi Hirano, Hitoshi Kagaya, Eiichi Saitoh, Yohei Otaka

**Affiliations:** ^1^ Faculty of Rehabilitation, School of Health Sciences, Fujita Health University, Toyoake, Japan; ^2^ Department of Rehabilitation Medicine I, School of Medicine, Fujita Health University, Toyoake, Japan; ^3^ Department of Rehabilitation, Fujita Health University Hospital, Toyoake, Japan

**Keywords:** spasticity, resistance, ankle joint, quantification, modified ashworth scale

## Abstract

**Background:** Despite recent developments in the methodology for measuring spasticity, the discriminative capacity of clinically diagnosed spasticity has not been well established. This study aimed to develop a simple device for measuring velocity-dependent spasticity with improved discriminative capacity based on an analysis of clinical maneuver and to examine its reliability and validity.

**Methods:** This study consisted of three experiments. First, to determine the appropriate motion of a mechanical device for the measurement of velocity-dependent spasticity, the movement pattern and the angular velocity used by clinicians to evaluate velocity-dependent spasticity were investigated. Analysis of the procedures performed by six physical therapists to evaluate spasticity were conducted using an electrogoniometer. Second, a device for measuring the resistance force against ankle dorsiflexion was developed based on the results of the first experiment. Additionally, preliminary testing of validity, as compared to that of the Modified Ashworth Scale (MAS), was conducted on 17 healthy participants and 10 patients who had stroke with spasticity. Third, the reliability of the measurement and the concurrent validity of mechanical measurement in the best ankle velocity setting were further tested in a larger sample comprising 24 healthy participants and 32 patients with stroke.

**Results:** The average angular velocity used by physical therapists to assess spasticity was 268 ± 77°/s. A device that enabled the measurement of resistance force at velocities of 300°/s, 150°/s, 100°/s, and 5°/s was developed. In the measurement, an angular velocity of 300°/s was found to best distinguish patients with spasticity (MAS of 1+ and 2) from healthy individuals. A measurement of 300°/s in the larger sample differentiated the control group from the MAS 1, 1+, and 2 subgroups (*p* < 0.01), as well as the MAS 1 and 2 subgroups (*p* < 0.05). No fixed or proportional bias was observed in repeated measurements.

**Conclusion:** A simple mechanical measurement methodology was developed based on the analysis of the clinical maneuver for measuring spasticity and was shown to be valid in differentiating the existence and extent of spasticity. This study suggest possible requirements to improve the quality of the mechanical measurement of spasticity.

## Introduction

Spasticity is characterized by a velocity-dependent increase in the tonic stretch reflex, which is clinically assessed as exaggerated tendon jerks (Lance, 1980). The increase in muscle tone due to spasticity restricts joint movements, resulting in an increase in the viscosity and stiffness of muscles and connective tissues (Singer et al., 2003; Brashear and Elovic, 2010; de Vlugt et al., 2010; Welmer et al., 2010; Kheder and Nair, 2012). In practice, clinical scales, such as the Ashworth Scale(AS) (Ashworth, 1964), Modified Ashworth Scale (MAS) (Bohannon and Smith, 1987), and Modified Tardieu Scale (MTS) (Boyd and Graham, 1999) are commonly used to quantify spasticity. These scales are easy to use, and previous studies support their reliability and validity (Bohannon and Smith, 1987; Allison et al., 1996; Gracies et al., 2010). Nonetheless, the findings of some studies contradict the reliability of these scales. For instance, Fleuren et al. reported the limited reliability of the AS; they showed that the inter-rater agreement was insufficient and that the rating was largely affected by whom the scale was rated on (Fleuren et al., 2010). Ansari et al. reported the poor inter-rater reliability of the AS and MAS (Ansari et al., 2006). Mehrholz et al. reported a poor-to-moderate inter-rater agreement after using the MAS and MTS to evaluate patients (Mehrholz et al., 2005). These results may be related to the difficulty in manually evaluating the differences in the resistance force (O’Dwyer et al., 1996), which is defined as the combination of hypertonic spasticity and increased viscoelastic properties of muscles and connective tissues (Dromerick, 2002; de Gooijer-van de Groep et al., 2013).

On the other hand, several studies have attempted to objectively evaluate spasticity using electrophysiological or kinetic methods, which may be more reliable in distinguishing velocity-dependent spasticity (Yanagisawa et al., 1993; Kagamihara et al., 1998; Rabita et al., 2005; Lorentzen et al., 2010; de Gooijer-van de Groep et al., 2013). However, there may still be room for improvement in the accuracy of these methods. For example, although most of the reports on quantification of spasticity with electrophysiological or kinetic methodology show a significant difference in values between healthy individuals and patients with spasticity, the detailed reports with raw values show an overlap of measurement values between patients with and without spasticity, patients and the healthy individuals, or patients with different levels of spasticity (Bakheit et al., 2003; Alibiglou et al., 2008; Lorentzen et al., 2010; de Gooijer-van de Groep et al., 2013), which is due to the large variance in measurement values. This may result in large minimal detectable changes, which may cast doubt on the usability of these methodology in real clinical practice.

There are several possible causes for this measurement variety. For example, inappropriate velocity for passive movement may affect the reliability of this methodology. Movement velocity seems to be important to detect spastic response of muscles; if too low, the spastic response may not be detected (Fujimura et al., 2022). Daily fluctuations in spasticity or the state of relaxation prior to measurement may also affect the measurement (Biering-Sørensen et al., 2006; Malhotra et al., 2009). Furthermore, there may also be unknown causes that affect the mechanical spasticity measurement.

To address this issue and to develop an objective methodology to detect the existence and extent of ankle spasticity more accurately, we began with an inductive approach and analyzed clinical maneuvers to assess spasticity. Accordingly, this study was conducted in three steps. First, the clinical procedures for evaluating spasticity were analyzed (Experiment 1). Second, a measurement device was assembled based on the findings of Experiment 1 to mechanically dorsiflex the ankle and measure the resistance force, and a preliminary validity study at several different joint movement velocities was subsequently conducted (Experiment 2). Third, the validity of the measurement for spasticity with the velocity that was best differentiated in Experiment 2 was assessed using a larger sample size. The test–retest reliability was also examined (Experiment 3).

## Materials and methods

### Experiment 1: Analysis of the procedures in the assessment of ankle plantar flexor spasticity by clinicians

#### Study group

Six physical therapists with 2–15 years of clinical experience were recruited as raters, and 15 patients with ankle plantar flexor spasticity were enrolled. Patients were recruited on an inpatient or outpatient basis at the Fujita Health University Hospital. The exclusion criteria were as follows: severe ankle contracture, pain related to ankle dorsiflexion, a history of cardiovascular or progressive neurological disease, or previous orthopedic surgery of the lower limb with spasticity. Out of the 15 enrolled patients, seven had cerebral hemorrhage, five had cerebral infarction, one had subarachnoid hemorrhage, one had a spinal cord injury, and one had a cerebral tumor. The mean age of the patients in the study group was 58 ± 17 years, and the median time after disease onset was 40 months (range, 1–109 months). The distribution of the MAS score for the ankle plantar flexors on the affected side was as follows: 0 (*n* = 1), 1 (*n* = 2), 1+ (*n* = 6), 2 (*n* = 4), and 3 (*n* = 2).

#### Assessment procedure

Patients were seated on a chair with the affected knee positioned at 60° of flexion while remaining relaxed. A twin-axis electrogoniometer (SG110; Biometrics Ltd., Newport, UK) was fixed to the ankle joint. The raters were asked to evaluate the existence and extent of velocity-dependent spasticity as they do in daily practice. The angular displacement of the ankle was measured using an electrogoniometer (sampling rate: 500 Hz). Each patient was assessed by two of the six raters. Each rater performed the assessment twice for each patient, and the data obtained in the second trial were included in the analysis. The procedures performed by the raters were video-recorded and analyzed by a panel of three clinicians (one physiatrist, two physical therapists), who were instructed to qualitatively analyze the procedures to develop a step-by-step description about a common testing procedure.

#### Analysis

Angular data were obtained using a low-pass-filtered electrogoniometer (10 Hz), and angular velocity was calculated as the difference between the point of maximum plantar flexion and the point of maximum dorsiflexion. The low-pass filter was set at 10 Hz because the ankle clonus is a set of involuntary and rhythmic muscle contractions at a frequency of 5–8 Hz (Boyraz et al., 2015). The normality of the distribution of angular velocity data was evaluated using the Shapiro–Wilk test.

### Experiment 2: Comparison of the resistance force against passive ankle dorsiflexion using a newly developed measurement device at several angular velocities

#### Study group

Seventeen healthy participants (4 men and 13 women; age, 24 ± 1 year; height, 159.5 ± 7.8 cm; weight, 53.1 ± 6.1 kg; maximum dorsiflexion range, 35 ± 7°) and 10 patients with stroke (relevant characteristics summarized in Table 1) were enrolled. Patients were recruited on an inpatient or outpatient basis at the Fujita Health University Hospital. Patients included in this experiment had sustained a stroke at least three months prior to enrollment in the study and were in a stable medical condition. The exclusion criteria were the same as that of Experiment 1.

**TABLE 1:** Patient demographics for Experiment 2.

Case	Sex	Age (years)	Height (cm)	Weight (kg)	Time after onset (months)	Diagnosis	Passive ankle D/F (°)	MAS of ankle plantar flexors	Achilles tendon reflex
1	M	61	167.0	62.0	10	CH	10	3	3+
2	M	52	170.0	81.0	60	CI	10	2	2+
3	M	73	172.0	60.0	49	CI	25	2	3+
4	M	65	165.0	69.0	71	CH	20	2	2+
5	M	26	179.0	90.0	5	CH	15	1+	2+
6	M	47	172.0	73.0	40	CH	25	1+	2+
7	M	76	168.0	65.0	109	CH	20	1+	1+
8	F	75	157.0	57.0	4	CI	20	1	2+
9	F	61	152.0	40.0	85	SAH	15	1	2+
10	F	49	158.0	59.0	79	CH	15	1	1+

F female, M male, CH, cerebral hemorrhage; CI, cerebral infarction; SAH subarachnoid hemorrhage, D/F dorsiflexion, MAS, modified ashworth scale.

#### Assessment procedure

**FIGURE 1:**
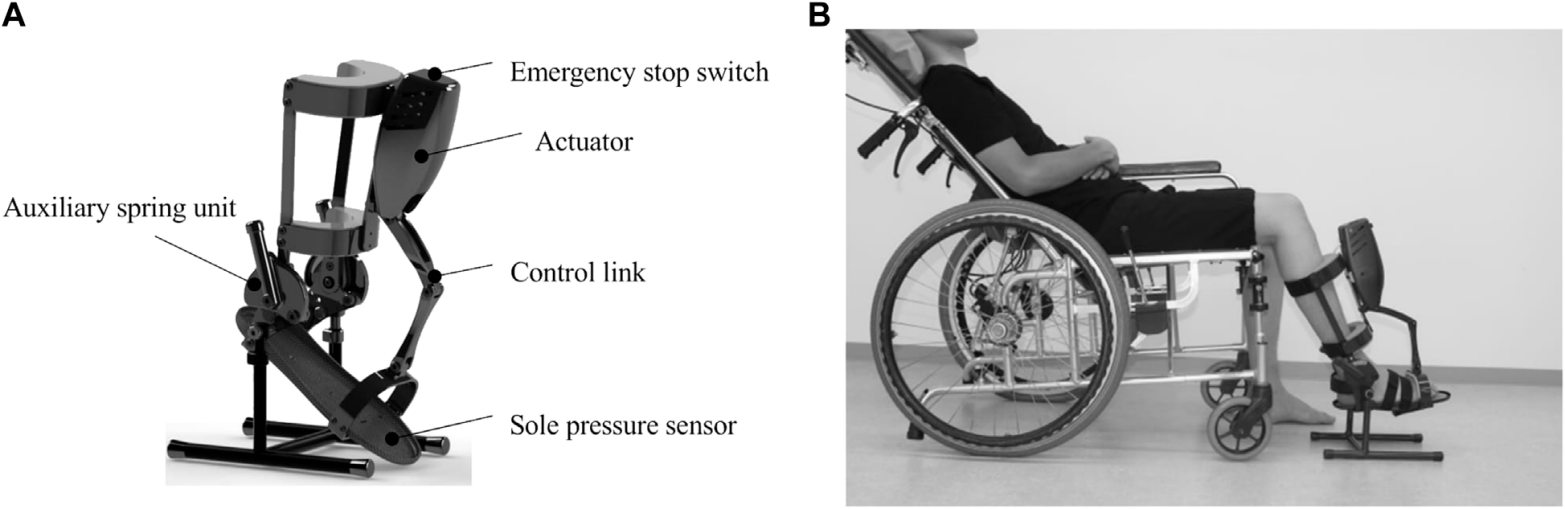
The main body of the developed device and the standard position for measurement.

We developed a measurement device for the passive dorsiflexion of the ankle joint at multiple fixed angular velocities. The device consisted of the following components: the main body, which included an actuator that was applied to the ankle to passively move its joint into dorsiflexion at a constant angular velocity (Figure 1); a computer with dedicated software that operated the main body of the device and recorded the angular data; and a power supply device, which was shared between the computer and the main body of the device. The servomotor passively rotated the foot plate of the device’s main body into ankle joint dorsiflexion at a constant velocity. The servomotor, actuator, and spring could yield a high angular velocity and an increased torque over a range of motion of 45° while being lightweight. The resistance force (N) to the passive ankle dorsiflexion movement was measured using a pressure sensor placed on the forefoot of the foot plate. The angular position of the ankle angle was sampled at a frequency of 1 kHz. The initial ankle angle was set at 25° of plantar flexion. The device was programmed to move as follows: 1) slowly dorsiflex to the maximum angle (5°, 10°, 15°, or 20°; the maximum angle within patients’ range of motion); 2) slowly plantar-flex to 25°; 3) dorsiflex to the maximum angle to measure spasticity at the pre-set velocity; 4) maintain for 3 s; and 5) slowly return to the position at 25° of plantar flexion. The angular velocities were set under three fast conditions (100°/s, 150°/s, and 300°/s), with a slow condition as the reference (5°/s). The height of the measurement unit was adjusted according to the length of the patients’ legs.

#### Measurement

The participants were asked to sit on a reclining wheelchair and to remain relaxed, with their neck in a neutral position. This posture was set to minimize the influence of postural reflexes, such as the tonic labyrinthine reflex and tonic neck reflex (Carr and Kenney, 1992), on the measured resistance to passive ankle dorsiflexion. The hip and knee were placed in 45° and 60° of flexion, respectively; this posture was chosen to avoid the influence of the length of the gastrocnemius muscle, which is a biarticular muscle. The size of the seat of the wheelchair was adjusted to include each of the patient’s thighs to reduce the influence of the weight of the thigh to the measurement. The height of the measurement device was adjusted depending on the leg length. Prior to measurement, the maximum dorsiflexion angle of the ankle was manually measured.

The device was applied to the right ankles of healthy participants and to the affected ankles of participants in the patient group. The resistance force as the device passively moved the ankle from 25° of plantar flexion to the maximum dorsiflexion angle at angular velocities of 5°/s, 100°/s, 150°/s, and 300°/s was recorded. The device was calibrated before every measurement. The order of angular velocity was randomly selected, with two trials being completed for each condition.

#### Analysis

The resistance force at rest was defined as the “0” reference. The mean peak resistance force was calculated from two trials at each angular velocity. These values were then compared between the patient and control groups using the Wilcoxon rank–sum test, and the patients were classified into subgroups according to the MAS score (0, 1, 1+, 2, and 3) for plantar flexor spasticity. Spearman’s rank correlation coefficient was calculated between the peak resistance force and MAS score. Each patient subgroup was compared to the control group using the Steel test (Steel, 1959), which is a multiple comparison rank sum test, used as a nonparametric version of Dunnet’s test (Dunnet, 1959).

### Experiment 3: Examination of the validity of the mechanical measurement of spasticity under the condition identified in experiment 2

#### Study group

To differentiate the existence and extent of spasticity, the sample size in each MAS subgroup was calculated using the sample-size calculating software G*Power (version 3.1.9.2) (Faul et al., 2007; Faul et al., 2009), with a power of 0.9, a significance level of 0.05, and the effect size as calculated in Experiment 2. To ensure sufficient power for comparisons, we ensured that the number of patients in each MAS subgroup exceeded the minimum sample size. Patients were recruited on an inpatient or outpatient basis at the Fujita Health University Hospital and were in stable medical condition. The exclusion criteria were the same as that of Experiment 1.

#### Assessment procedure and measurement

The measurement device developed in Experiment 2 was used. The measurement was performed at the best angular velocity identified in Experiment 2 to differentiate spasticity. The movement of the device was programmed in the same manner as in Experiment 2, except for a single high angular velocity. The sampling rate was 1 kHz. The measurement was conducted using the same procedure as outlined in Experiment 2.

#### Analysis

The resistance force at rest was defined as the “0” reference. The mean peak resistance force was calculated from two trials performed at each angular velocity. As in the MTS (Boyd and Graham 1999), the differences between the responses to the fast and slow passive ankle movements could reflect the velocity-dependent muscle response against passive ankle movements. The difference between the peak resistance force at 5°/s and the highest angular velocity was subsequently calculated. Receiver operating characteristic (ROC) curves were employed to determine the optimal diagnostic cut-off values that discriminate controls from patients with spasticity (excluding MAS 0 patients). The criteria for the cut-off value was set by the Youden’s index. The sensitivity and specificity of the measurement values were evaluated. Differences in values were compared between the patient and control groups using the Wilcoxon rank-sum test. The patients were then classified into subgroups according to the MAS score (0, 1, 1+, 2, and 3) for plantar flexor spasticity. The correlation between the extent of spasticity, assessed using the MAS, and the measurement values was determined using Spearman’s rank correlation test. Differences between all pairs of the control group and patient subgroups were assessed using the Steel-Dwass test (Dwass 1960; Steel 1960), which is a nonparametric version of Tukey’s test (Tukey, 1949). Similarities between the two trials were examined using a Bland–Altman plot (Altman, 1991; Atkinson and Nevill, 1998) for each test. The limits of agreement (LOA) were calculated as the mean difference ±1.96 standard deviation (SD) of the difference and were presented in Bland–Altman plots. In addition, 95% confidence intervals (CIs) were calculated for the upper and lower LOA. Fixed bias was computed as the average difference between the 1st and 2nd measurements, statistically checked by the 95% CI of the mean differences between two values ( $\bar{d}$ ). Fixed bias was indicated if the 95% CI of $\bar{d}$ did not include zero. Proportional bias was expressed as the correlation coefficient between the difference and the average of the 1st and 2nd measurements. A Bland–Altman analysis was performed, and the 95% LOAs were calculated using the BlandAltmanLeh R package (Lehnert 2015). The minimum detectable change at 95% CI (MDC_95_) was calculated as follows:

MDC95=1.96×√2×SEM

where SEM is the standard error of measurement (Faber et al., 2006).

All other statistical analyses were performed using JMP version 13 (SAS Institute Inc., Cary, NC, USA), with *p* < 0.05 considered to be statistically significant.

#### Ethical considerations

This study was approved by the Ethics Committee of Fujita Health University (HM17-456, CRB418003), and all participants provided written informed consent for their participation in this study. All the methods in this study were performed in accordance with the relevant guidelines and regulations. This study was registered in UMIN-CTR (UMIN000026305) on 25 February 2017, jRCT (jRCTs042180044) on 21 November 2018, and UMIN-CTR (UMIN000040472) on 21 May 2020.

## Results

### Experiment 1

From the qualitative video analysis of procedures performed by the clinicians, the clinician panel described the common testing procedure of raters in the spasticity rating session. The process consisted of the following five steps: 1) holding the patients’ ankle; 2) slow maximum stretch to a dorsiflexion position; 3) slowly putting back the ankle to a neutral or mild plantar flexion position; 4) rapid dorsiflexion of the ankle to the maximum dorsiflexion position; and 5) slowly returning the ankle to a neutral position. Furthermore, the panel pointed out that the clinicians commonly placed their hands below the calf and lifted the lower legs during measurement; this was interpreted by the panel as the movement to cancel the weight of the thigh and lower legs.

The angular velocities of each rater are presented in Table 2. The physical therapists applied an angular velocity of 268 ± 77°/s (mean ± SD) to evaluate the degree of spasticity in the ankle plantar flexors. The angular velocities used were normally distributed (*p* = 0.205).

**TABLE 2:** Angular velocity of the ankle joint (°/s) according to the raters’ manual assessment.

	Case															
	1	2	3	4	5	6	7	8	9	10	11	12	13	14	15	Mean
Rater A	189	−	269	182	169	−	−	−	−	−	−	−	−	−	306	223
B	215	−	−	−	−	266	−	−	274	−	−	−	255	313		265
C	−	162	−	−	−	229	409	296	−	−	−	−	−	−	408	301
D	−	−	365	−	−	−	332	−	−	414	352	−	−	361	−	365
E	−	−	−	−	304	−	−	202	−	243	−	205	150	−	−	221
F	−	231	−	182	−	−	−	−	208	−	244	301	−	−	−	233
Mean	202	196	317	182	236	248	370	249	241	328	298	253	202	337	357	268

### Experiment 2

A measurement device was developed to replicate the common testing procedure described in Experiment 1. The motion consisted of slow dorsiflexion to the maximum dorsiflexion and angle, as well as slow plantar flexion, followed by dorsiflexion to the maximum dorsiflexion angle at the pre-set velocity for measurement. Additionally, to reduce influence of the weight of the legs we employed the following two approaches; first, the supporting rod of the measurement unit was made adjustable to tailor the height of the measurement unit to the length of patients’ lower legs. Second, the size of the patients’ seat was adjusted to cover each patient thigh to reduce the influence of the weight of the thigh. Further details on the setting and motion of the device are provided in the Methods section.

**FIGURE 2:**
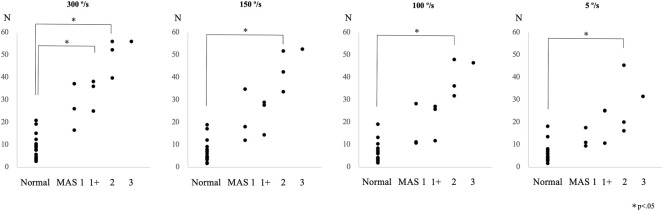
Peak resistance force values at angular velocities of 300°/s, 150°/s, 100°/s, and 5°/s are shown for the control and the patient subgroups, classified according to the Modified Ashworth Scale (MAS) score in Experiment 2.

The peak resistance force in the control and patient groups, including all subgroups, was found to be 8.9 ± 5.5 N (mean ± SD) and 38.4 ± 13.5 N (effect size *d* = 2.9; *p* < 0.01) at an angular velocity of 300°/s, 6.9 ± 5.0 N and 31.7 ± 14.4 N (effect size *d* = 2.3; *p* < 0.01) at 150°/s, 6.3 ± 4.5 N and 27.9 ± 13.6 N (effect size *d* = 2.1; *p* < 0.01) at 100°/s, and 6.2 ± 4.2 N and 21.4 ± 11.1 N (effect size *d* = 1.8; *p* < 0.01) at 5°/s, respectively. The peak resistance force between the control group and each MAS subgroup is shown in Figure 2. The rank correlation coefficient between the resistance force and the MAS score was as follows: 300°/s, r_s_ = 0.84 (*p* < 0.01); 150°/s, r_s_ = 0.80 (*p* < 0.01); 100°/s, r_s_ = 0.81 (*p* < 0.01); and 5°/s, r_s_ = 0.78 (*p* < 0.01).

### Experiment 3

We calculated the minimum sample sizes of the subgroups from Experiment 2 to investigate the difference between them. We calculated the difference between the control group and each subgroup as 7 for the control group and MAS 1, 3 for MAS 1+, and 3 for MAS 2. A total of 24 healthy participants (13 men and 11 women; age, 23 ± 1 year; height, 164.3 ± 9.4 cm; weight, 54.4 ± 8.3 kg; maximum dorsiflexion range, 31 ± 6°) and 32 patients with stroke were enrolled. Of the 32 patients, 18 had cerebral hemorrhage, 11 had cerebral infarction, and 3 had subarachnoid hemorrhage. The distribution of the MAS score for the ankle plantar flexors on the affected side was as follows: 0 (*n* = 3), 1 (*n* = 10), 1+ (*n* = 10), 2 (*n* = 7), and 3 (*n* = 2).

In the control group, the peak resistance force was 17.2 ± 10.2 N at 5°/s and 22.6 ± 9.6 N at 300°/s, and the difference between the two conditions was 5.4 ± 2.6 N (mean ± SD). In the patient group, the peak resistance force was 29.3 ± 16.8 N at 5°/s and 53.5 ± 21.1 N at 300°/s, and the difference was 24.3 ± 12.0 N.

**FIGURE 3:**
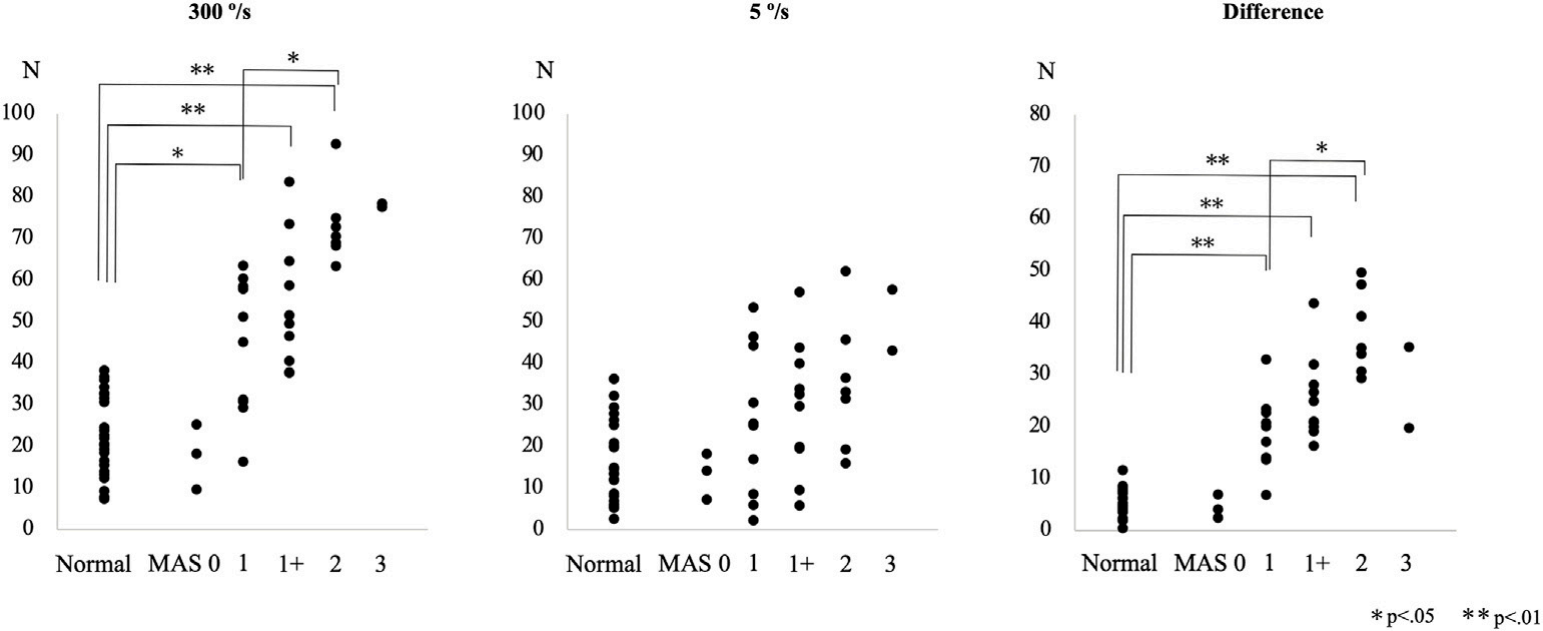
Peak resistance force values at angular velocities of 300°/s and 5°/s and their differences are shown for the control and the patient subgroups, classified according to the Modified Ashworth Scale (MAS) score, in Experiment 3.

**FIGURE 4:**
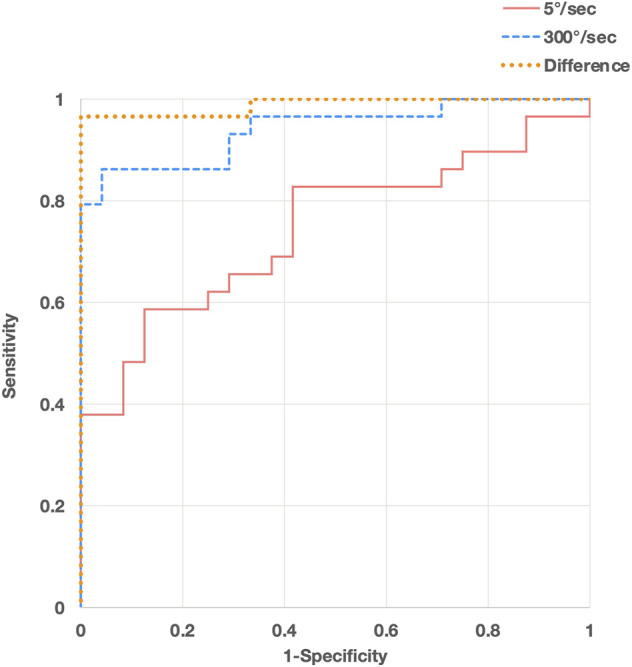
Receiver operating characteristic (ROC) curve analyses of peak resistance force values at angular velocities of 300°/s (blue, dashed line) and 5°/s (red, solid line) and their differences (orange, dotted line) for discriminating patients with spasticity from controls.

The difference in the resistance force between the control group and each MAS subgroup is shown in Figure 3. The rank correlation coefficient between the resistance force and the MAS score was as follows: 5°/s, r_s_ = 0.50 (*p* < 0.01); 300°/s, r_s_ = 0.84 (*p* < 0.01); difference value (300°/s - 5°/s), r_s_ = 0.88 (*p* < 0.01). The optimal diagnostic cut-off values that discriminate controls from patients with spasticity, excluding MAS 0 patients, were assessed using ROC analysis. The ROC curves of the peak resistance force at 5°/s and 300°/s and the difference between them revealed that the areas under the curve (AUC) were 0.744, 0.941, and 0.988 (*p* < 0.001), with an optimal cut-off value of 29.7, 37.8, and 13.7 N, respectively. Sensitivity and specificity were 58.6 and 87.5% at 5°/s, 86.2 and 95.6% at 300°/s, and 96.6 and 100% with the difference value, respectively (Figure 4).

**FIGURE 5:**
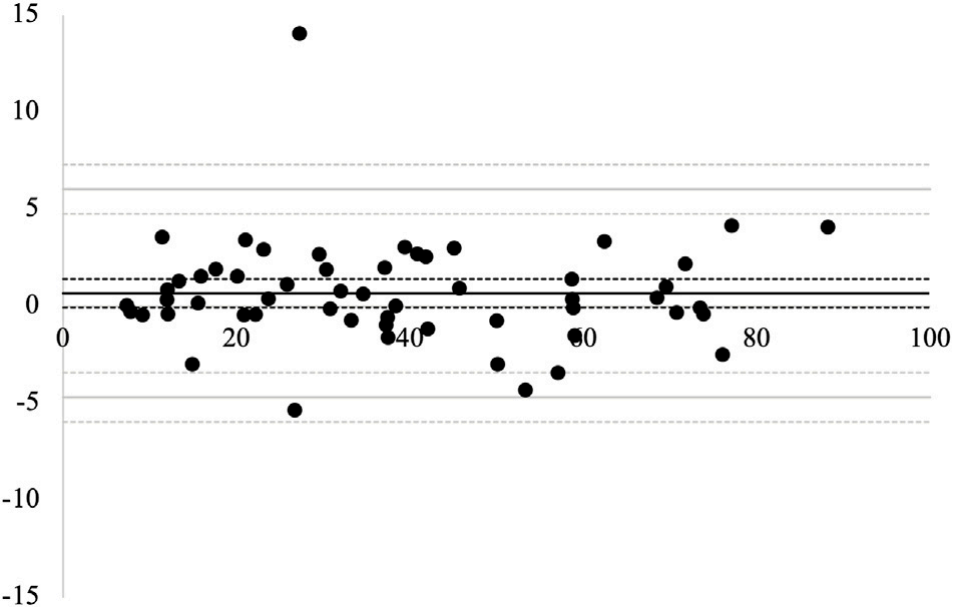
Bland–Altman plots for the 1st and 2nd measurements. The solid black line represents the average difference between the two measurements. The solid gray lines represent the limits of agreement (LOA). The dotted lines represent 95% confidence intervals.

The Bland–Altman plot for the agreement between the two measurement trials is presented in Figure 5. The mean difference between the 1st and 2nd measurements was 0.67 (95% CI, −0.06–1.41). There was no significant difference between the 1st and 2nd measurement values. The LOA between the measurements were between −4.68 (95% CI, −5.95 to −3.41) and 6.03 (95% CI, 4.76–7.30). No proportional bias was observed between the 1st and 2nd measurements (*r* = −0.03, *p* = 0.80). The MDC_95_ value was 5.36.

## Discussion

In this study, the clinical procedures for spasticity assessment were visually analyzed using an electrogoniometer (Experiment 1). Additionally, a preliminary validation of the measurement device developed according to the results of Experiment 1 and a comparison of measurement conditions were conducted (Experiment 2), which indicated that an angular velocity of 300°/s was the best for detecting spasticity. Subsequently, the validity and reliability of measurement with the determined condition were tested using a larger sample size in Experiment 3, which showed high test–retest reliability and known-groups validity between patients with spasticity and healthy controls, and concurrent validity with MAS. The measurement at an angular velocity of 300°/s significantly correlated with MAS and detected the difference between the control group and MAS1 subgroup, as well as the MAS1 and 2 subgroups.

The results of Experiment 1 indicated that in the clinical evaluation of spasticity, the clinicians moved the patients’ ankle at an angular velocity of 268 ± 77°/s. This was faster than the velocities employed in previous studies on the mechanical measurement of spasticity, which ranged from 25°/s to 211°/s (Singer et al., 2003; Rabita et al., 2005; Lorentzen et al., 2010; de Gooijer-van de Grop et al., 2013; Tomita et al., 2014). As reported by previous studies, spastic responses to passive joint movement are velocity-dependent (Rabita et al., 2005). Thus, the fast velocity employed by clinicians may contribute to an increase in sensitivity in detecting spasticity. The necessity of this fast angular velocity was further tested in Experiment 2 using a measurement device developed based on observations of the clinical maneuver. Among the velocities of 100°/s, 150°/s, and 300°/s, 300°/s was found to best discriminate the patient group from the control group and to best differentiate between different degrees of spasticity classified using the MAS score. Altogether, these results strongly support the importance of angular velocity in improving the sensitivity of the mechanical measurement of spasticity.

In addition, the video analysis of the measurement procedure in Experiment 1 revealed the small tips that could possibly affect the accuracy of the measurement. For example, the clinicians often added a preparatory slow stretching movement prior to the rapid dorsiflexion of the angle for the measurement. This might have potentially affected the sensitivity of the measurement of velocity-dependent spasticity, as the elastic component of ankle resistance, which can be reduced by passive stretching (Bressel and McNair, 2002; Singer et al., 2008), influences the measurement of resistance force. In addition, the clinicians also commonly held the calf and lifted the lower legs to cancel the weight of the thigh and lower legs, thus further influencing the measurement value. Therefore, we added the preparatory motion in the measurement of stretching and adjusted the seat length and height of the device to minimize the influence of the weight of the thigh. The necessity of each of these additional tips in the measurement of spasticity may have to be further clarified; nonetheless, the high discriminative capacity of this methodology may support the potential benefits of this clinical practice-based approach in the development of methodology for mechanical measurement.

In Experiment 3, the validity of the measurement at an angular velocity of 300°/s was further tested using a larger sample size. The results indicated that this measurement condition can clearly discriminate the spastic patients from control and further, the differences between the subgroups could be detected with this measurement condition; the differences between the control group and MAS 1 subgroup, as well as the MAS 1 and 2 subgroups, were significant. These results support the sensitivity of this measurement methodology at a high angular velocity of 300°/s and the difference value with 5°/s in detecting the existence of spasticity and differences in extent. Previous studies showed the validity of the mechanical measurement of spasticity in joints, such as the ankle and wrist, with a significant correlation with clinical scales, such as MAS or MTS (Alibiglou et al., 2008; Andringa et al., 2019). However, the strength of correlations with clinical scales and the discriminative capacity to differentiate the existence and extent of spasticity were still limited. In this study, we reported a high correlation between the measurement values and MAS score and the detection of the existence of mild spasticity (MAS 1) and difference in mild and moderate spasticity (MAS1 and 2). These results support the clinical feasibility of the measurement methodology developed in this study. In contrast, no significant differences in the measurement at a velocity of 5°/s were identified between the MAS subgroups. As the low angular velocity of 5°/s was insufficient to induce a stretch reflex response (Lamontagne et al., 1997; Lorentzen et al., 2010), measurements at 5°/s would reflect the resistance to movement due to non-neural factors, such as the elastic properties of muscles and connective tissues (Xu et al., 2020). Therefore, the comparison between fast and slow conditions is considered to mainly reflect the neural component of spasticity (Lindberg et al., 2011). In fact, the value representing the difference between the fast and slow conditions exhibited better discriminative ability compared to the value for either condition, effectively detecting spasticity with very high sensitivity and specificity. Although there are a number of studies on the mechanical measurement of spasticity, few have reported adequate sensitivity and specificity levels (van der Velden et al., 2022). The high sensitivity and specificity obtained in this study are encouraging for further development and clinical implementation of mechanical devices for spasticity measurement. The MDC_95_ value, which is the minimum value considered a real change beyond the range of measurement error, was 5.36 N, which seemed sufficient to detect spasticity given the large mean difference in measurement values between the control and patient groups: 12.1 N at 5°/sec and 20.9 N at 300°/sec.

The results obtained in this study may further contribute to the detailed understanding about spasticity. There have already been several attempts to separate the neural and non-neural components of spasticity in previous studies (Phillips et at., 2004; Usuba et al., 2007; Lindberg et al., 2011; de Gooijer-van de Groep et al., 2013) that were based on biomechanical modeling, using mechanical measurement under several different velocity conditions. In this study, we succeeded in discriminating different degrees of spasticity *via* a simple measurement with mechanically controlled passive ankle movement at fast and slow velocities, without conducting a sophisticated component analysis with biomechanical modeling. Further combination of this measurement methodology with biomechanical modeling may further improve the accuracy of the estimation of neural and non-neural components of spasticity, promoting a more detailed understanding about the mechanism of spasticity.

### Study limitations

This study has some limitations that should be considered. First, the analysis of the clinical maneuver was performed in a single hospital with a limited number of clinicians; thus, the findings may not be generalized as a common maneuver used by clinicians. In addition, although the developed methodology based on clinical maneuver was presented with high discriminative capacity of the spasticity, the necessity of each condition determined by the analysis of maneuver was not determined. Therefore, some of the employed conditions can be irrelevant to the discriminative capacity of the methodology. The necessity of each condition should be determined with further detailed studies. Even though, considering the high discriminative capacity achieved in this study, clues to improve the accuracy of the mechanical measurement of spasticity were at least provided with this inductive approach. Second, electromyographic analysis was lacking, which would have confirmed the presence or absence of a stretch reflex response during maneuvers, thereby supporting the analysis of the contribution of the velocity-dependent component of joint resistance. Our study focused on the relationship between the speed of joint movement and the resistance force, which is simple to measure. However, future studies combining torque measurement with this methodology and electromyography would aid in further understanding the mechanism of the response against joint movement. Third, due to technical limitations, we could not test at a higher velocity than 300°/s. Therefore, we cannot exclude the possibility that a higher velocity better distinguishes spasticity. Further technical development may facilitate the understanding of the velocity-response relationship in the measurement of spasticity. Fourth, some of the stroke patients with spasticity in this study had contracture of their plantar flexion, which may affect the measurement results, increasing the values obtained at both at 5°/sec and 300°/sec. However, the maximal dorsiflexion angle in measurement was set at the maximal value that does not exceed the patients’ range of motion, and the influence of this restriction in the range of motion is expected to be minimal. In addition, the use of the value representing the difference between measurements at 5°/sec and 300°/sec may help reduce the influence of the contracture. Finally, the reliability and validity of the MAS has been controversial (Mehrholz et al., 2005; Ansari et al., 2006; Fleuren et al., 2010), which may affect the credibility of the results in the present study. Validation using scales with greater reliability, such as the modified MAS (Ansari et al., 2006; Ghotbi et al., 2011), should be considered in future studies. Nevertheless, our approach’s significant ability for identifying spastic patients at least supports the validity of using mechanical measurement for detecting spasticity.

## Conclusion

In this study, a simple objective mechanical measurement methodology was developed to detect the existence and extent of ankle spasticity by analyzing clinical maneuver. The results of this series of the experiments indicated that sufficiently fast movement during measurement is important in the mechanical measurement of spasticity. In addition, the developed device demonstrated sufficient test–retest reliability and clinical validity. These results should support the realization of a simple and accurate measurement of spasticity in daily clinical practice.

## Data Availability

The raw data supporting the conclusion of this article will be made available by the authors, without undue reservation.
